# DNA enrichment and tagmentation method for species-level identification and strain-level differentiation using ON-rep-seq

**DOI:** 10.1038/s42003-019-0617-x

**Published:** 2019-10-10

**Authors:** Łukasz Krych, Josué L. Castro-Mejía, Laura M. Forero-Junco, Daniel N. Moesby, Morten B. Mikkelsen, Morten A. Rasmussen, Maciej Sykulski, Dennis S. Nielsen

**Affiliations:** 1 Food Microbiology and Fermentation, Department of Food Science, University of Copenhagen, 1958 Frederiksberg C, Denmark; 2 GenXone S.A., 60-476 Poznań, Poland; 3 Computational Biology and Microbial Ecology, Department of Biological Sciences, Universidad de los Andes, Bogotá, Colombia; 4 Chemometrics and Analytical Technology, Department of Food Science, University of Copenhagen, 1958 Frederiksberg C, Denmark; 5 COPSAC, Copenhagen Prospective Studies on Asthma in Childhood, Herlev and Gentofte Hospital, University of Copenhagen, Copenhagen, Denmark

**Keywords:** High-throughput screening, DNA sequencing, Bacterial techniques and applications, Microbiology techniques, Nanopores

## Abstract

Despite the massive developments within culture-independent methods for detection of microorganisms during the last decade, culture-based methods remain a cornerstone in microbiology. Yet, the problem of rapid, accurate and inexpensive identification of bacterial isolates down to species/strain level remains unresolved. We have developed a new method for bacterial DNA enrichment and tagmentation allowing fast (<24 h) and cost-effective species level identification and strain level differentiation using the MinION portable sequencing platform (ON-rep-seq). DNA library preparation for 96 isolates takes less than 5 h and ensures highly reproducible distribution of reads that can be used to generate strain level specific read length counts profiles (LCp). We have developed a pipeline that by correcting reads error within peaks of LCp generates a set of high quality (>99%) consensus reads. Whereas, the information from high quality reads is used to retrieve species level taxonomy, comparison of LCp allows for strain level differentiation.

## Abstract (web summary)

Łukasz Krych et al. uses MinION portable sequencing platform to develop a fast and cost-effective method for bacterial DNA enrichment and tagmentation (ON-rep-seq). Their method allows species-level identification and strain-level differentiation of 288 isolates on a single flow for less than $2 per sample at very high speed.

## Introduction

Culture-dependent methods remain indispensable in detailed identification of bacteria. Yet, successful typing of bacteria down to species/strain level remains not fully resolved^1^. Several promising technologies and methodologies for solving the problem have been proposed, but with a variable success. Generally, fast and cost-effective methods are not accurate enough, while those that are more accurate are also more laborious and/or expensive. Methods based on 16S ribosomal RNA (rRNA) gene sequencing are amongst the most universal, yet species-level resolution cannot always be reached^2^. More complex molecular tools that are able to reach strain-level resolution, such as pulse-field gel electrophoresis (PFGE), repetitive extragenic palindromic PCR (Rep-PCR), multilocus sequencing typing (MLST), or matrix-assisted laser desorption/ionization-time-of-flight mass spectrometer (MALDI-TOF MS), are hampered by one or several drawbacks that include low speed/throughput, limited databases, no taxonomic information, laborious procedure, or high equipment cost^3–5^.

The present gold standard for strain-level bacterial identification is full genome sequencing. Optimally this approach combines information from high-throughput, short, good quality reads with lower throughput, poor quality but long reads^6^. However, this approach is far from being cost effective, and the data analysis and interpretation is far from trivial^7–9^.

The portable DNA-sequencing platform MinION by Oxford Nanopore Technologies (ONTs) offers an attractive tool with a potential to tackle the task of species/strain-level identification^10^. Unfortunately, ONTs still deal with two critical problems: relatively high error rate at the base level and lower throughput compared to technologies offered by, for example, Illumina^10^. We propose a DNA enrichment method that to a large extent have solved both these pitfalls by combining an optimized version of Rep-PCR with a consecutive dual-stage Rep-PCR-2 step during which sample-specific barcodes are incorporated.

Repetitive extragenic palindromic sequences in bacterial genomes were first described in the genomes of *Escherichia coli* and *Salmonella* in 1984 by M.J. Stern^11^. A decade later J. Versalovic used interspersed repetitive sequences as a binding site for primers developing Rep-PCR^12^. Amplicons varying in length (from few dozen base pairs (bp) to few kilo base pairs (kbp)) separated with electrophoresis create a genomic fingerprint that has been proven many times to have species and in some setups also strain-level discriminative resolution of bacteria^13^. Only 5 years later Rep-PCR was described as one of the most reproducible and commonly used method for species- and strain-level discernment^14^, and numerous applications of the method have been reported in many fields, including food processing, food safety, environmental microbiology, and medicine^15–19^. Despite the immense progress in DNA-sequencing technologies, Rep-PCR is still a commonly used technique in many research groups mainly due to the low cost of the analysis and basic laboratory equipment needed^20^. However, the low running costs comes with a price of highly laborious and time-consuming procedures involving 3–5 h PCR, 3–5 h electrophoresis, and complicated, tedious, and potentially error-prone fingerprint data analysis. However, most importantly, classical Rep-PCR only allows for bacterial discrimination but not direct identification^21^.

We are presenting a new bacterial DNA enrichment method for Oxford Nanopore sequencing called ON-rep-seq. The method exploits an optimized version of Rep-PCR for reproducible amplification followed by a dual-stage Rep-PCR-2 step allowing tagmentation of up to 96 samples in one reaction. Furthermore, we have developed a pipeline utilizing the information from the generated sequences at three levels: (i) generation and comparison of isolate-specific read length count profiles (LCps); (ii) detection of peaks in each LCp followed by within-peak correction of the random single base error; (iii) species-level taxonomy assignment using corrected consensus reads. The method has been tested on 38 different bacterial species- and three strain-level groups successfully identifying all bacteria down to the species-level and discriminating strains with a sensitivity that is at least similar to a whole-genome sequencing (WGS)-based approach.

## Results

### Rep-PCR-enriched library generates highly reproducible LCp

**Fig. 1:**
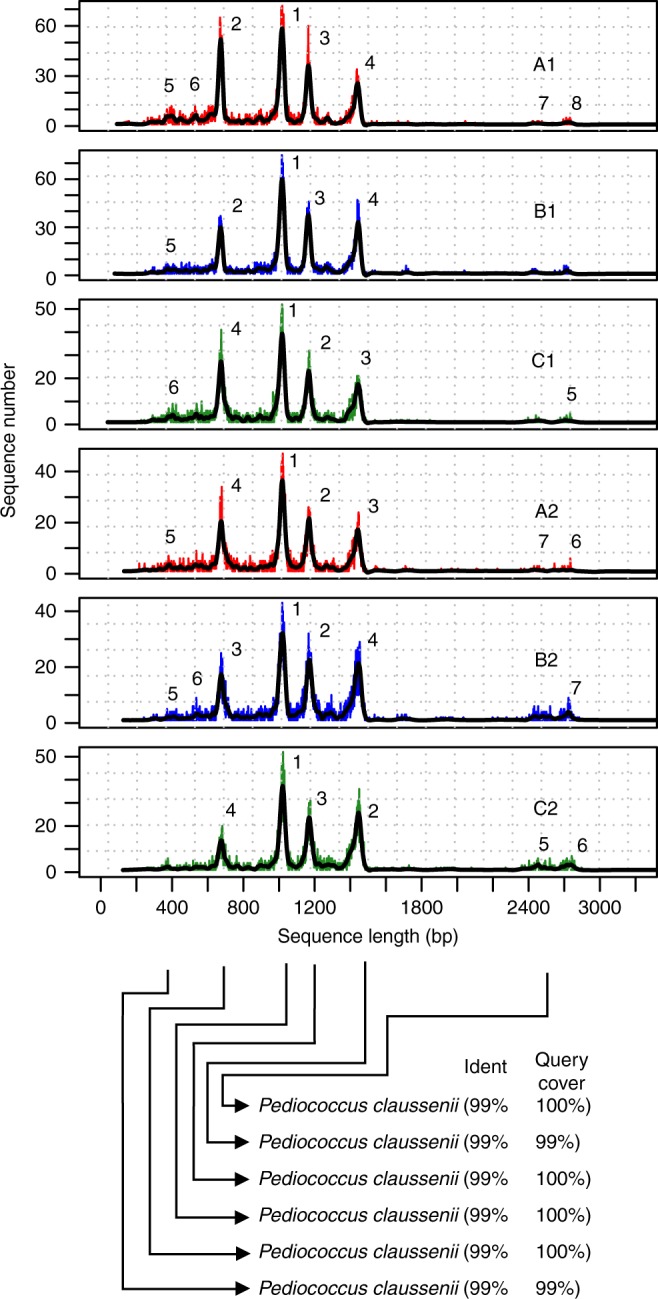
*Pediococcus claussenii* Oxford Nanopore Technology-based rep-PCR amplicon sequencing (ON-rep-seq) read length count profiles (LCps). LCps were generated from ON-rep-seq of *Pediococcus claussenii*. All technical replicates of *P. clausenni* profiles show high level of similarity across three consecutive sequencing runs (red, blue, and green for run A, B, and C, respectively) and two technical replicates in each run. The retrieved sequences matching the length of a corresponding peak were subjected for correction using Canu and consensus sequences were verified using blastn. For all profiles six to eight high-quality reads could be generated, each with >99% similarity to the reference genome of *P. clausenni*. The number above each peak indicates the peak detection sensitivity, with 1 being the most evident. The minimum number of reads within the peak needed for reads correction is 50

Similar to Rep-PCR gel-based fingerprints, sequenced Rep-PCR products can be transformed into LCps, which is a function of reads length and abundance. The shape and position of peaks is highly reproducible in all technical replicates across first two sequencing runs (Fig. 1, Supplementary Figs. 1 and 2), indicating that the barcode sequences do not affect the shape or the position of the peaks during Rep-PCR-2. Yet, as explained below, we observed a minor run effect in the third consecutive run resulting in shifted distribution of short/long reads.

### Corrected reads from LCp provide detailed identification

A single band on a gel (or peak in LCp) of a Rep-PCR profile will contain mainly representatives of the same amplicon, which would allow for base accuracy correction using tools such as, for example, Canu^22^. With that assumption, we have developed a pipeline operating in three steps: strain-specific LCp generation and comparison, within-peak reads correction, and peak’s consensus sequence annotation. The pipeline generated on average 10 high-quality consensus reads for each isolate (max = 26; min = 3; SD = 4) with mean length of 1 kbp (max = 3.6 kbp; min = 0.3 kbp; SD = 0.6 kbp). The number of reads used for correction within a peak (cluster size) varied from 50 to 2400 (mean = 254; SD = 246).

Subjecting the set of corrected reads for each sample to metagenomic classifier (kraken2 or centrifuge) allowed for unambiguous classification of all bacteria down to the species and subspecies level (Table 1). The average sequence similarity of corrected reads from strain validated with Illumina sequencing (*S. enterica* serovar Typhimurium C5) reached 99.4% (BLAST; min = 98.3%, max = 100%, SD = 0.5%). Among the isolates tested are, for example, *Lactobacillus casei* and *Lactobacillus paracasei* subsp. *paracasei* known to be indistinguishable based on 16S rRNA gene sequence comparison or *Lactococcus lactis* subsp. *cremoris* that cannot be distinguished from *Lactococcus lactis* subsp. *lactis*. All these strains were unambiguously discriminated using ON-rep-seq. Two bacterial species: *Bacteroides thetaiotaomicron* and *Lactococcus lactis* subsp*. cremoris* were tested in pairs from different culture collections resulting in all cases in highly reproducible LCp (Supplementary Fig. 1).

**Table 1:** Results of bacterial isolates identification with ON-rep-seq

Bacterium and kraken2 retrieved classification	Strain	Average number of corrected reads per sample	Average number of corrected bases
*Akkermansia muciniphila*	DSMZ 22959	9	4166
*Bacillus cereus*	15	5	8279
*Bacillus cereus*	NVH 38	7	7648
*Bacillus licheniformis*	LMG19409	7	8603
*Bacillus subtilis*	In-house strain	9	4707
*Bacteriodes cellulosilyticus*	DSM 14838	9	7775
*Bacteriodes eggerthii*	DSM 20697	8	5366
*Bacteriodes finegoldii*	DSM 17565	9	6720
*Bacteriodes intestinalis*	DSM 17393	13	11,264
*Bacteriodes thetaiotaomicron*	ATCC 29148	9	8597
*Bacteroides thetaiotaomicron*	DSM 2079^T^	12	8158
*Bacteriodes vulgatus*	LMG 17263	11	8875
*Bacteroides fragilis*	DSM 2151	10	6765
*Bifidobacterium adolescentis*	DSM 20083	14	7729
*Bifidobacterium animalis*	DSM 10140	16	10,306
*Bifidobacterium bifidum*	LMG 11041	9	9635
*Bifidobacterium breve*	DSM 20091	14	10,388
*Bifidobacterium catenulatum*	LMG 11043	16	13,216
*Bifidobacterium longum* subsp. *longum*	LMG 13196	16	7808
*Bifidobacterium longum* subsp. *infantis*	DSM 20090	17	11,134
*Bifidobacterium pseudocatenulatum*	LMG 10505	17	12,818
*Escherichia coli*	DSM 1058	14	12,897
*Lactobacillus acidophilus*	LMG 9433^T^	5	7121
*Lactobacillus amylovorus*	DSM 20531^T^	10	10,966
*Lactobacillus brevis*	GGUC30670T	8	12,532
*Lactobacillus casei*	DSM 20011^T^	13	10,238
*Lactobacillus fermentum*	DSM20052^T^	9	12,409
*Lactobacillus paracasei* subsp. *paracasei*	NCFB151T	11	13,292
*Lactobacillus paracasei*	In-house strain	12	8728
*Lactobacillus plantarum*	ATCC14917^T^	7	8023
*Lactobacillus plantarum*	DSM 20174^T^	7	11,459
*Lactobacillus rhamnosus*	DSM 20021^T^	12	12,199
*Lactobacillus sakei* subsp. *sakei*	DSM 20017^T^	9	3267
*Lactococcus lactis* subsp. *cremoris*	MG 1363	6	7124
*Lactococcus lactis* subsp. *cremoris*	Wg2	6	9094
*Leuconostoc mesenteroides* subsp. *mesenteroides*	DSM 20343^T^	9	9469
*Listeria monocytogenes*	EGDe	8	6598
*Listeria monocytogenes*	L028	7	8209
*Listeria monocytogenes*	N53-1	7	5967
*Listeria monocytogenes*	12067	7	8557
*Listeria monocytogenes*	42222/180	9	6378
*Pediococcus claussenii*	DSM 14800^T^	6	10,290
*Pediococcus pentosaceus*	DSM 20336^T^	8	6713
*Salmonella enterica* subsp. *enterica* serovar Oranienburg	0112F	13	7316
*Salmonella enterica* subsp. *enterica* serovar Typhimurium	U292	9	12,155
*Salmonella enterica* subsp. *enterica* serovar Typhimurium	4//74	9	9380
*Salmonella enterica* subsp*. enterica* serovar Typhimurium	C5	8	5971
*Streptoccocus thermophilus*	S0	11	10,764

Subjecting the set of corrected reads for 48 bacterial isolates to metagenomic classifiers (kraken2 or centrifuge) allowed for unambiguous annotation of all bacteria down to the species and subspecies level

### Strain-level differentiation using LCp

**Fig. 2:**
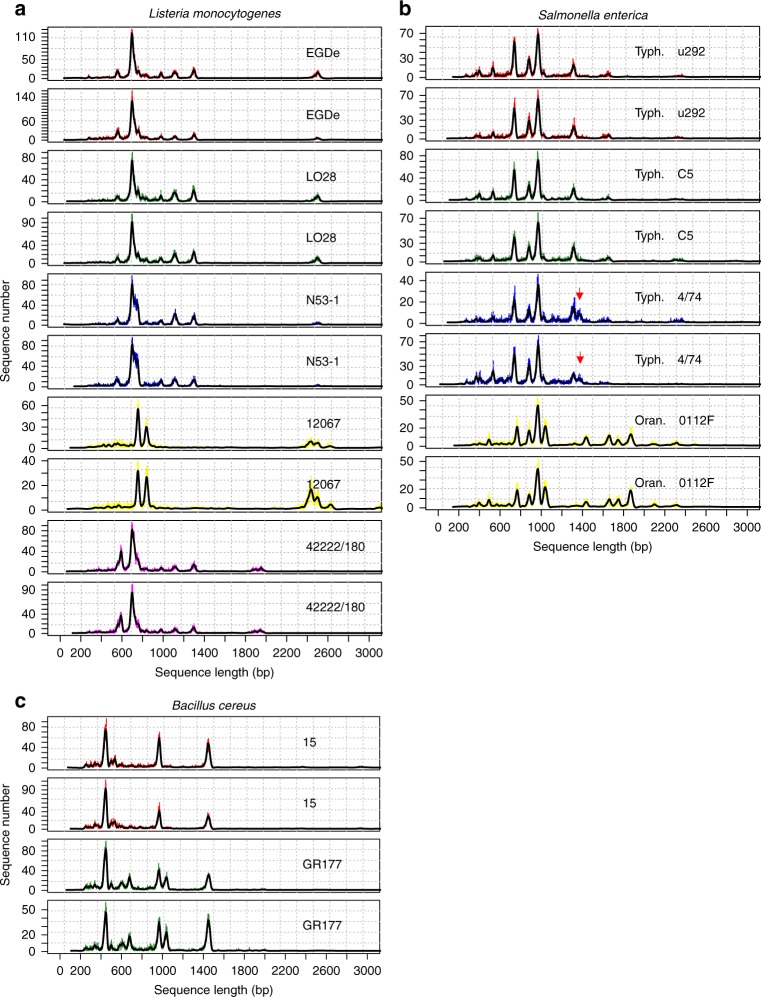
Examples of strain-level differentiation using LCp comparison. **a**–**b** Oxford Nanopore Technology-based rep-PCR amplicon sequencing (ON-rep-seq) of five *L. monocytogenes* (**a**), four *S. enterica* (**b**), and two *B. cereus* (**c**) strains was used to generate read length count profiles (LCp). All bacterial LCps were produced in duplicates. Consensus sequences from corrected peaks of all 22 samples allowed for unequivocal species- and subsbecies-level identification. Comparison of LCps revealed four different profiles among the *L. monocytogenes* species. Strains EGDe and LO28 gave highly similar profiles, indicating high level of genetical relationship between these two strains (**a**), which was confirmed by Illumina-based shotgun sequencing (orthoANI = 99.9%). Similarly C5 and u292 strains of *S. typhimurium* showed the same profiles (orthoANI = 99.9%), while two other strains could be classified as different (**b**). The red arrows indicate additional peak distinguishing the 4/74 strain from u292 and C5 that was shown to have a prophage origin. The presence of additional peaks in the LCp of GR177 strain allowed for unambiguous differentiation between the two *B. cereus* strains (**c**)

**Fig. 3:**
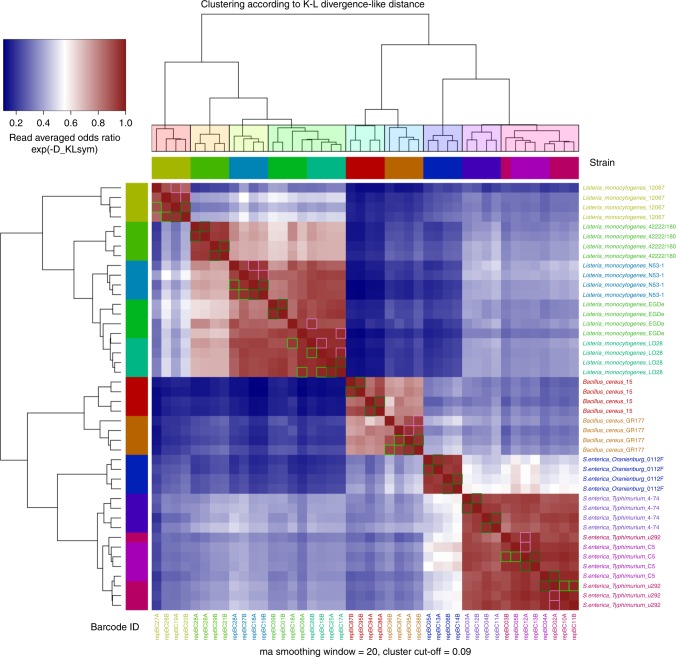
Row/column clustering according to Ward.D2 hierarchical clustering on D_KLsym distance. Heatmap showing similarity (10^(-D_KLsym)), and clusters according to cut-off = 0.09. Analysis of five *Listeria monocytogenes*, two *Bacillus cereus*, and four *Salmonella enterica* serovar Typhimurium strains allowed for species-level differentiation in all cases and for strain-level differentiation in 8 out of 11 cases. Notably, the presence of the additional peak allowed for unambiguous differentiation of 4/74 from C5 and u292, which was not possible using OrthoANI and MLST analysis based on WGS data. Strain labels colors according to accepted strain similarity derived from visual inspection of profiles in agreement with clustering colors at selected cut-off

Five *Listeria monocytogenes*, four *Salmonella enterica* (three serovar Typhimurium and one serovar Oranienburg), and two *Bacillus cereus* strains have been used to evaluate the method for strain-level discrimination. We have developed an algorithm (LCpCluster.R) estimating the level of similarity between the pairs of LCp generated by the ON-rep-seq. Among five *L. monocytogenes* strains four unique profiles were identified (Figs. 2a and 3). Strains EGDe and LO28 generated identical profiles (Figs. 2a and 3).

No SNP variants could be detected when comparing consensus sequences of corresponding peaks of all technical replicates. WGS data have been used to estimate the genetic similarity between EGDe and LO28 strains. The average nucleotide identity (OrthoANI) index between these two genomes reached 99.9%. Also, the k-mer distribution comparison indicated high level of similarity between the two strains (Jaccard distance for shared k-mers = 0.0005). Finally, *L. monocytogenes* MLST schemes mapped against EGDe and LO28 found only one differing locus (*dapE*) out of seven tested (Supplementary Table 1). These results imply a high level of genetical similarity between the two strains that requires a specific approach to ensure differentiation.

Among four *S. enterica* strains, LCpCluster.R recognized three unique profiles (Figs. 2b and 3). Serovar Typhimurium strains u292 and C5 showed the same ON-rep-seq LCp with no SNP variants in corresponding peaks. WGS comparison of these two strains revealed high OrthoANI reaching 99.9% and high k-mer distribution similarity (Jaccard distance for shared k-mers = 0.0005). *Salmonella enterica* MLST schemes mapped against genomes of u292 and C5 also showed the same alleles profiles (Supplementary Table 1). This implies that *S. enterica* strains u292 and C5 could not be straightforwardly distinguished based on their genome using both methods.

Interestingly, serovar Typhimurium strain 4/74 that presented similar LCp to u292 and C5, yet with a clear additional peak in the position ~1370 bp (Figs. 2b and 3), reached OrthoANI above 99.9%, high k-mer distribution similarity (Jaccard distance for shared k-mers <0.0003), and had the same MLST profile compared to u292 and C5. In this particular example, ON-rep-seq presented higher discrimination power over OrthoANI, k-mer distribution analysis, and MLST analysis based on WGS data. Further investigation of the peak at position ~1370 bp disclosed that the consensus sequence presented high similarity (blast identity 1372/1384 bp; 99.1%) to SopEΦ prophage. Moreover, this sequence could only be found in the draft genome of the 4/74 strain (blast identity 1371/1384 bp; 99.1%), but not in any of the remaining *S. enterica* strains.

Finally, the two *B. cereus* strains generated clearly distinctive LCp and were classified as different strains (Figs. 2c and 3). The LCp.Cluster results showing grouping according to Ward.D2 hierarchical clustering on D_KLsym distance of all 48 isolates in four technical replicates from first two runs are given in Supplementary Fig. 2.

### The theoretical throughput reaches over 1000 isolates

To validate the method, two R9.4.1 flow cells were benchmarked for the maximum possible output generated. The first benchmarked flow cell generated in total over 2.6M reads (after quality control and demultiplexing). See Supplementary Table 2 for details. In the first four consecutive runs, each lasting 4 h, intertwined with the flow cell washing steps and storage for minimum 24 h enough data was generated to successfully demultiplex and identify 4 × 96 bacterial profiles on a single flow cell. The last run generated 0.22M reads, which was enough to detect and correct sequences of 94 out of 96 samples.

The second flow cell generated in total 2.49M reads, respectively, 1, 0.56, and 0.87M for first (4 h), second (4 h), and third (12 h) run. See Table Supplementary Table 2. All three runs of the second flow cell generated enough data to successfully analyze 96 bacterial profiles. To verify the minimum number of reads necessary to analyze all samples, the data have been iteratively subsampled and subjected to the analysis with a receiver operating characteristic (ROC) curves to quantify trade-off between pairwise same/not-the-same strain discrimination dependent on clustering cut-off. Throughout the analysis, it was noticed that within-strain variance was larger than between-strain variance in cases of small differing features in the latter, and the disproportion of short reads vs. long reads in the former case (the observation verified by sample mean read length regression vs. sample read count; Supplementary Fig. 3). This disproportion was attributed to the third sequencing run on the reused flow cell, hence the latest repC run was omitted from the cluster analysis and most of ROC curves analysis that follows.

Clustering on different data sets were compared: all, wo.rep*C (without the third consecutive run: repC), 2, 10, 20, and 50% subsamples. wo.rep*C performed best most of the time, although random fluctuations in 50, 20, and 10% subsamples overperformed occasionally at single data points. Subsampling to 50 and 20% (avg. #reads/sample 4326 and 1730) performed very similarly to full samples (avg. #reads/s 8652), while 10% subsamples performed worse, although still reasonably good, while 2% subsamples (avg. #reads/s 173) performed much worse, although relevant information is still present and retrievable even with such a small read length sample (Supplementary Fig. 4a–f).

The flow cell benchmarking results showed that 20% of generated reads (avg. #reads/sample 1730) were already sufficient to analyze all samples. Notably, the number of isolates that could be analyzed simultaneously on a single flow cell will ultimately depend on the number and position of peaks in LCp (for strain-level comparison). Nonetheless, our data demonstrate that the theoretical throughput of the R9.4.1 flow cell ranges between 960 and 1440 isolates depending on the sequencing run performance (~1.5 to ~2.5 M reads, respectively).

## Discussion

The process of fast and accurate bacterial identification, subtyping, and strain-level differentiation is of high importance in epidemiology, to recognize infection outbreaks, determine its source, or follow highly virulent nosocomial pathogens. It is also desired in the food industry to validate quality and safety and to investigate microbiologically complex communities like many fermented foods. For the past three decades the most commonly used and standardized methods became molecular techniques based on DNA analysis^14^. Since first described in 1994 Rep-PCR targeting REP and/or repetitive intergenic consensus (ERIC) regions became a widely used method of DNA typing^12,17^. Its discriminatory power has been shown multiple times to be superior to many other typing methods including ribotyping^12,23^, multilocus enzyme electrophoresis^24,25^, and also biochemical characterization^26^. Rep-PCR was often shown to have similar or slightly lower discriminatory power than PFGE, but was always considered a less laborious and cheaper solution^27–29^. Among several Rep-PCR options, (GTG)5-PCR have reported to be the most robust^30^. Despite well-documented strain-level discrimination power, the main pitfall of Rep-PCR is without a doubt its inability for taxonomic identification, without additional analysis such as 16S rRNA gene sequencing, which requires extra laboratory work and increases time and cost of the analysis. Moreover, such strategy relays entirely in the discriminatory power of 16S rRNA gene that does not always allow for species-level identification.

The massive leap in DNA-sequencing methods made within the past decade heralded the inevitable decline of many old-fashioned DNA fingerprint-based typing methods. A single HiSeq X instrument (IIlumina) has a capacity to sequence about 35,000 average size bacterial genomes with 100 times coverage in a single run (Illumina.com). Yet, notwithstanding the immense potential, this technology is still not meant for fast, routine, and cost-effective typing of bacteria. It is mainly due to the high equipment cost, low flexibility requiring collection of multiple samples (from dozens to thousands depending on the platform), relatively long runtime, and complex data analysis. The portable, USB powered MinION offered by ONTs is so far the cheapest (~$1000) sequencing platform on the market. Its main advantage besides the price is the possibility to generate ultra-long reads with the longest ones crossing 1 Mb. Nonetheless, there are two main reasons why ONTs have not yet become the first choice of a sequencing strategy in many laboratories. First is the relatively high basecalling error rate of a single DNA molecule and second, a relatively low throughput compared to many other platforms^10^. Our method have largely solved these two hindrances, allowing ONTs to be exploited for accurate, large scale, and detailed identification of bacteria. Highly reproducible amplification of regions flanked by REP elements not only solved the main problem with sequencing redundancy one needs to deal during WGS but also enabled single base error correction owing it to its random nature. ON-rep-seq offers the well-documented discrimination power of the DNA fingerprint analysis, but also for the first time full access to the hidden information within each band DNA sequence in quality crossing 99% accuracy. Since each isolate composes on average of 10 corrected consensus reads with an average length of 1 kb, this information can be used for highly accurate taxonomic identification. Even if one of the reads would not find a hit in a database, there are still several others to ensure classification. As shown here, all 48 isolates have been accurately assigned to the species and subspecies level. Also, for the first time users will be able to easily determine contaminations in case one or several peaks would turn out to belong to another organism. Lastly, by reducing the number of samples and increasing the coverage one could achieve even higher accuracy of the consensus sequence what could be used to assess the presence of SNPs in profiles of closely related strains. This might be an additional source of previously unknown discrimination repository of Rep-PCR in some unique cases.

We have demonstrated here that ON-rep-seq successfully identified and differentiated between *Listeria monocytogenes* and *Salmonella enterica* serovar Typhimurium strains. Both species are of special epidemiological importance and model organisms for host–pathogen infection^31,32^. Rep-PCR was previously recommended method for subtyping of *L. monocytogenes* and *S. enterica* with a similar discrimination power of PFGE or RAPD^29,33^. The only two undistinguished pairs of strains were *L. monocytogenes* EGDe from LO28 and *Salmonella enterica* C5 from u292. Paired comparison of their genomes revealed high level of similarity (OrthoANI >99.9%) further confirmed with the MLST, indicating that genetic diversity between these two strains could be allocated in SNPs. Regrettably, none of these SNPs were found by comparing sequence within the peaks. Although ON-rep-seq cannot discriminate between strains that differs solely with SNPs, it can be used for fast and cost-effective screening of multiple isolates to select those of identical profiles that should be subjected for deep sequencing saving resources, money, and time.

Interestingly, ON-rep-seq was shown to be superior to traditional WGS analysis in distinguishing between *S. enterica* u292 and 4/74. The OrthoANI between these two strains reached >99.9% with identical MLST profiles. This makes it very challenging to differentiate between u292 and 4/74 at the strain level using WGS^34,35^. However, comparison of ON-rep-seq-based LCp allowed for clear and unambiguous differentiation between the two strains. The peak allowing this distinction was shown to be a mobile element with high similarity to a prophage. It was previously demonstrated that large fractions of genetic variation in *Salmonella* strains is allocated in variable genomic regions and islands that encompass phage insertions^36^.

Presented in this work, barcodes enable accurate tagmentation of 96 isolates, but our data demonstrate that even about 1000 barcodes could be used on a single R9 flow cell. We have benchmarked two R9.4.1 flow cells to estimate the maximum possible output and cost per isolate. Since the flow cell price ranges between $475 and $900 (depending on the bundle offer), the sole cost of sequencing assuming highest output would range between $0.40 and $0.75. It is important to mention that the maximum data output will vary depending on the flow cell viability that may be affected by multiple washing steps. Furthermore, it was demonstrated herein that consecutive usage of the flow cell may be a source of an increased run effect. Therefore, the best performance of ON-rep-seq could be achieved if, for example, 96 × 10 barcodes were used in a single run lasting for maximum time (48 h). It seems however that the new gadget offered by ONTs called Flongle, promised to be released this year, could be the most optimal and user-friendly solution for ON-rep-seq (nanoporetech.com). Flongle is an adapter for MinION with one-quarter throughput of a R9 flow cell but price not crossing $100. This means that up to 3 × 96 isolates could be analyzed in a single run for about $0.35 per sample. Naturally, the user could then choose to sequence less isolates but ensure even better coverage.

In summary, we present here the DNA enrichment and barcoding method called ON-rep-seq (from: Oxford Nanopore-based Rep-PCR-based sequencing), which in combination with ONT-sequencing platforms allows for highly cost-effective, bulk screening of bacterial isolates with species and strain-level resolution. We believe that ON-rep-seq has a potential to become a modern standard molecular-based method with multiple applications in research, industry, and medicine. By sharing it to other users, we are looking forward for thorough validation of many more bacterial species, optimization of sequencing protocols, and pipelines. We hope that conjoined effort of multiple users will also allow for the development of ON-rep-seq consensus reads database facilitating in the even faster and simplified identification.

## Methods

### Wet laboratory

*Rep-PCR-1*: Bacterial genomic DNA was extracted using GenElute^TM^ Bacterial Genomic Kit (Sigma Life Science, Darmstadt, Germany) according to the manufacturer’s instructions. In total, 48 isolates represented by 38 different bacterial species were subjected for the analysis in duplicates in each of the three runs giving six technical replicates per isolate. The barcode order was shifted during preparation of each library to ensure that every technical replicate is tagged with different barcode sequence. Three strains of *Salmonella enterica*, five *Listeria monocytogenes*, and two *Bacillus cereus* strains have been used to evaluate the ability of the method for strain-level differentiation. The detailed list of bacteria used for the analysis is given in Table 1, while ON-rep-seq LCp is given in Supplementary Fig. 1. The Rep-PCR reaction mix contained 5 μl PCRBIO HiFi buffer (5×), 0.25 μl of PCRBIO HiFi Polymerase (PCR Biosystems Ltd, London, UK), 4 μl of (GTG)5 primers (5 μM), 1 μl of DNA (~20 ng/μl), and nuclease-free water to a total volume of 25 μl. The Rep-PCR thermal conditions were optimized as follows: denaturation at 95 °C for 5 min; 30 cycles of 95 °C for 30 s, 45 °C for 1 min, and 62 °C for 4 min, followed by final elongation at 72 °C for 5 min. It is important to note that several polymerases have been tested in order to shorten the elongation time without compromising the longest amplicons. With current settings, the PCR takes <3 h on SureCycler 8800 (Agilent, CA, USA).

*Barcoding by dual-stage Rep-PCR-2*: We have designed 96 ONT-compatible barcodes (Supplementary Table 3) with 15 bp spacer separating ONT motor protein adapter from the barcode sequence and (GTG)5 pairing region. The spacer was added to ensure higher tolerance for the low quality at the beginning of the sequence entering the pore and thus higher recovery of barcode sequence. At the same time the spacer sequence was designed to prevent creations of stem loops in relatively long primers during low temperature annealing step. The Rep-PCR reaction mix contained 12 μl of PCRBIO UltraMix (PCR Biosystems Ltd, London, UK), 2 μl of corresponding repBC primer (10 μM), 1 μl of PCR product from Rep-PCR-1, and nuclease-free water to a total volume of 25 μl. Incorporation of ONT-compatible adapters (Supplementary Table 3) was performed using dual-stage PCR where first 3 cycles provide optimal annealing of (GTG)5 regions, while next 10 cycles allow for best hybridization of full adapters in consecutive cycles: denaturation 5 min; 3 cycles of 95 °C for 30 s, 45 °C for 1 min, and 62 °C for 4 min, followed by 10 cycles of 95 °C for 30 s, 65 °C for 1 min, and 72 °C for 4 min, and final elongation at 72 °C for 5 min.

*Library preparation and ONT-sequencing*: After Rep-PCR-2 samples were pooled using 10 μl of each sample. Note that samples were not pooled in equimolar concentration due to expected differences in length of amplified regions between the samples. However, it is advisable to verify the DNA concentration with Qubit® dsDNA BR Assay Kit (Life Technologies, CA, USA) for the quality control of the Rep-PCR-2 step. The measurement was performed with Varioskan Flash Multimode Reader (Thermo Fischer Scientific, MA, USA). Fluorescence was measured at 485/530 nm.

The pooled library was cleaned with AMPure XP beads (Beckman Coulter Genomic, CA, USA) in volumes 100:50 μl, respectively. The bead pellet was washed with 80% ethanol and re-suspended in 100 μl of nuclease-free water. The bead washing step was added to shift the proportion of short to long reads that is multi-template PCR-specific feature and to remove primer dimers. The pooled and bead-purified library was measured with Qubit® dsDNA HS Assay Kit (Life Technologies, CA, USA), and 66 ng of library was used as an input to the End-prep step in 1D amplicon by ligation protocol (ADE_9003_v108_ revT_18Oct2016) with one adjustment: 80% ethanol instead of 70% was used for all washing steps.

To validate our method, we have benchmarked two R9.4.1 flow cells for the maximum possible output generated. First flow cell was used 10 days after the delivery in five consecutive runs, each lasting 4 h, intertwined with the flow cell washing steps and storage for minimum 24 h (QC 1347 active pores). Second flow cell was used 44 days after the delivery in three consecutive runs lasting, respectively, 4, 4, and 12 h (to collect maximum amount of data from declining flow cell; QC 1105 active pores). After each run the flow cell was washed according to the manufacturer’s instructions and a new library was prepared and loaded. In order to evaluate the possibility of barcode-specific amplification during Rep-PCR-2 step, all samples received different barcode in consecutive sequencing runs. The data from the first benchmarked flow cell were used solely to test the optimal concentration of DNA needed and viability of the flow cell, while data from the second flow cell are presented herein and can be downloaded from SRA NCBI repository (#SUB4333515).

### Data analysis

*Data collection, base calling, demultiplexing, and trimming*: Data were collected using Oxford Nanopore software: MinKnow 1.10.23 (https://nanoporetech.com). The amount of data collected in both R.9.4 flow cells is listed in Supplementary Table 2. Guppy 2.1.3 Basecalling Toolkit was used to base call raw fast5 to fastq (https://nanoporetech.com). Porechop v.0.2.2 was used for adapters trimming and samples demultiplexing (https://github.com/rrwick/Porechop). Porechop settings together with the list of custom adapters (adapters.py) compatible with oligos given in Supplementary Table 3 are available at (https://on-rep-seq.readthedocs.io/en/latest/index.html). The script allows for demultiplexing up to 96 barcodes and trimming of: ONT adapters, custom spacers, and tandem repeats of (GTG)*n*.

*Correction and base location of peaks*: Peaks are identified in LCp expressed as sequencing length (*x*-axis) by number of reads (*y*-axis) by fitting local third-order polynomials in a sliding window of size 1/50 of the *x*-span across the *x*-axis, followed by calculation of the first- and second- order derivatives. The position of a peak is identified at the *x*-axis where the first derivative is zero and the second derivative is negative. Only peaks with intensity higher than baseline, defined as a moving boxcar (zero-order polynomial) in a broad window (4 times the size of the window used for calculation of the derivative) are used for further analysis. The identified peaks are ordered based on the height, and a representative fragment is used for database matching.

*Reads correction within a peak*: Sequences containing quality scores (fastq files) resolved within each peak were retrieved using Cutadapt v1.15^37^, and corrected with Canu v1.6^22^ using the following parameters: genomeSize = 5k, minimumReadLength = 200, correctedErrorRate = 0.05, corOutCoverage = 5000, corMinCoverage = 2, and minOverlapLength = 50. The corrected reads were sorted by length and clustered with cluster_fast from VSEARCH^38^, using the following options: -id of 0.9, -minsl of 0.8, -sizeout, and min_cons_pct of 20. The purpose of this step is to detect structural sequence variants of similar length. Subsequently, consensus sequences were sorted by size (coverage) and those with a minimum coverage size of 50× were kept for downstream analyses.

*Classification*: Centrifuge 1.0.3^39^ and kraken2^40^ metagenomic classifiers were tested for classification of corrected reads. Although both classifiers performed with similar accuracy that allowed adequate annotation of all bacteria, kraken2 is recommended especially when novel bacteria are expected.

*Comparison of LCp*: The identification of a good distance measure on read LCp was approached by considering them as approximating samples of their underlying sampling distributions. Ideally one would like to understand processes involved in signal peaks and noise formation, thus a priori distributions could be postulated, and later optimized for profiles posteriors. Primarily, empirical discrete length distributions were smoothed with window moving average (ma). Selection of ma window size was done by computing the average jitter of all profiles: an average number of times when profile’s discrete derivative changes sign (change to 0 was counted as 0.5). From mean jitter plot ma window size was selected to 20, the point of the lowest second derivative, after which second derivative stabilized closely around 0, meaning the information (jitter) loss due to increasing of window size became relatively low and more constant (Supplementary Fig. 4g, h). Next with each LCp assigned was a ma smoothed and probability-normalized distribution profile Dp.

Stability problems around #reads(*i*) = Dp(*i*) = 0 are avoided by considering a mixture of ma smoothed Dp and the uniform read length distribution (in a considered range 150–3000 bp) with proportions (0.99, 0.01). The distance between two samples, read length-based, was defined as a function of LCp_1, Dp_1, LCp_2, and Dp_2. One natural approach was to consider the probability of sampling LCp_1 from Dp_2; however, for the distance to be comparable between samples of different read counts, it needed to be normalized by total read count. Resulting is the following logarithmic formula:

$$D_{\mathrm{nprob}}(LCp_1\|Dp_2)=-\sum_i\frac{LCp_1(i)}{\#LCp_1}\,\log_{10}{Dp_2(i)}\,=\,-\sum_iP_{LCp_1(i)}\,\log_{10}{Dp_2(i)},$$

The above formula is, however, not centralized because the distance of a sample to itself is not 0, but it is rather equal to sample’s smoothed entropy. Centralization of this distance yields distance very similar to Kullback–Leiber divergence of probabilities, which is proposed for the distance between LCp, as follows:

$$D_{\mathrm{KL}}(LCp_1\|Dp_2)=-\sum_iP_{LCp_1(i)}\,\log_{10}\frac{Dp_2(i)}{Dp_1(i)},$$

In the following clustering analysis, we use the symmetrized version:

$$D_{\mathrm{KLsym}}(LCp_1,LCp_2)\,=\,\frac{D_{\mathrm{KL}}(LCp_1\|Dp_2)+D_{\mathrm{KL}}(LCp_2\|Dp_1)}{2}\,.$$

*Analysis of D_KLsym distance on bacterial LCp*: Validation of KL-based distance on LCp by hierarchical clustering was performed on sequencing results where clusters were compared with down-to-strain sample labels. To promote clusters with low variance around centroids Ward.D2 clustering method was selected and performed with modified heatmap3 R library. LCp data samples from the same strain are expected to form spherical cluster due to randomness. On the other hand, deviations from sphericity are likely due to correlated signal from additional peak(s) on distributions sampled from, which makes them reasonable candidates for new cluster(s). This fact is reflected in Ward’s clustering method results, when compared to complete (single) clustering methods, which performed comparably/slightly worse (significantly worse), on the whole data, but much worse and less consistent when run on several subsamples (50, 20%) of data; ROC curve was characteristic considered as a measure of performance. Figure 3 shows clusters recovered with cut-off = 0.09 where all *L. monocytogenes*, *B. cereus*, and *S. enterica* strains with clearly visible feature peaks were properly clustered.

**Fig. 4:**
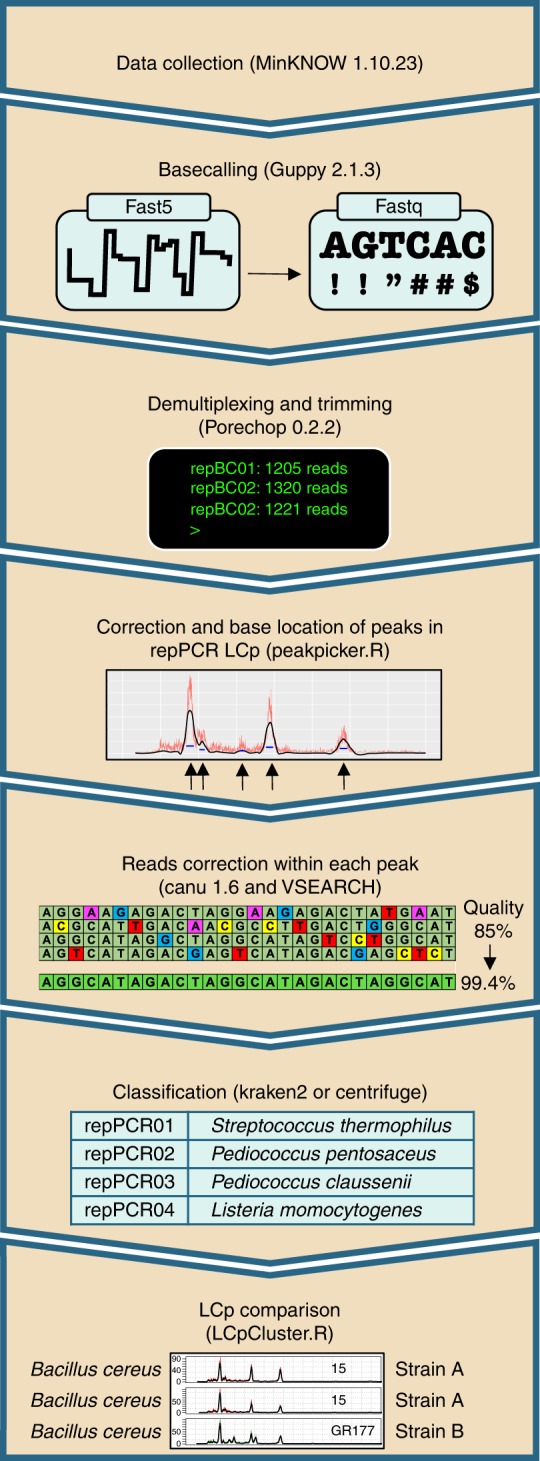
ON-rep-seq pipeline overview. The schema describing pipeline that allows processing of the raw Oxford Nanopore Technology-based rep-PCR amplicon sequencing (ON-rep-seq) data. After initial basecalling, demultiplexing (separating according to barcodes) the fastq files are used to generate read length count profile (LCp) based on sequences length distribution. Subsequently, reads within each peak are clustered with VSEARCH, corrected with Canu, followed by taxonomy classification using improved quality reads. Finally, the traces can be compared to estimate strain-level relatedness between pairs of LCp

*ON-rep-seq analysis toolbox*: We have developed a pipeline called ON-rep-seq toolbox available through GitHub repository allowing user-friendly analysis from raw fastq files to taxonomy classification and LCp clustering. The overview of the pipeline is given in Fig. 4.

*WGS data analysis*: Complete or draft genomes *L. monocytogenes* EGDe (#NC_003210.1) and LO28 (#AARY02000001.1-2001127.1); *S. enterica* serovar Typhimurium ST4/74 (#CP002487.1) and u292 (#ERR277220) were downloaded from public databases.

Comparisons between genomes were conducted using OrthoANI^8^ and the K-mer Analysis Toolkit^41^.

For strain C5, DNA was subjected to library preparation (Nextera XT Kit, following the manufacturer’s procedures) and sequencing on Illumina NextSeq platform. High-quality reads (>95% quality and minimum size of 50 nt using Trimmomatic v.0.35^42^ were de-convoluted from phiX174 controls reads (-id: 0.97, -query_cov: 0.97) and dereplicated using VSEARCH^43^. Subsequently, reads were assembled into contigs using Spades v.3.5.0^44^.

Contigs with a minimum size of 10,000 bp generated for C5 strain, and in addition to the publicly available U292 and 4/74 putative genomes, were subjected to MLST analysis on the CLC Genomics Workbench v11.1 using a minimum alignment length of 400 bp and high level of alignment stringency.

*Salmonella enterica* MLST schemes (internal fragments and their alleles) hosted at PubMLST.org were mapped against genomes of U292 (#ERR277220) and 4/74 (#CP002487.1), as well as the assemble contigs of C5 strain included in this study.

### Reporting summary

Further information on research design is available in the Nature Research Reporting Summary linked to this article.

## Supplementary information

Supplementary Information

Description of additional supplementary files

Supplementary Data 1

Reporting Summary

## Data Availability

Fastq files after demultiplexing (for each isolate and each technical replicate separately) can be downloaded from SRA NCBI repository (#SUB4333515) https://www.ncbi.nlm.nih.gov/sra. Raw fast5 and fastq files are available upon reasonable request (contact: Ł.K.).

## Code availability

The updated version of ON-rep-seq pipeline is available through GitHub repository: https://on-rep-seq.readthedocs.io/en/latest/index.html. The version of the ON-rep-seq pipeline described in this manuscript (v1.2) is stored at Zenodo repository (10.5281/zenodo.3384842).

## References

[1] Marx V (2016). Microbiology: the road to strain-level identification. Nat. Methods.

[2] Janda JM, Abbott SL (2007). 16S rRNA gene sequencing for bacterial identification in the diagnostic laboratory: pluses, perils, and pitfalls. J. Clin. Microbiol..

[3] Hrabák J, Chudácková E, Walková R (2013). Matrix-assisted laser desorption ionization-time of flight (MALDITOF) mass spectrometry for detection of antibiotic resistance mechanisms: from research to routine diagnosis. Clin. Microbiol. Rev..

[4] Rodriguez M (2015). Discriminatory indices of typing methods for epidemiologic analysis of contemporary *Staphylococcus aureus* strains. Medicine.

[5] Sandrin TR, Goldstein JE, Schumaker S (2013). MS profiling of bacteria at the strain level: a review. Mass Spectrom. Rev..

[6] Miller, J. R. et al. Hybrid assembly with long and short reads improves discovery of gene family expansions. *BMC Genomics*10.1186/s12864-017-3927-8 (2017). 10.1186/s12864-017-3927-8 PMC5518131 28724409

[7] Carlisle EM (2012). Murine gut microbiota and transcriptome are diet dependent. Ann. Surg..

[8] Lee I, Kim YO, Park SC, Chun J (2016). OrthoANI: an improved algorithm and software for calculating average nucleotide identity. Int. J. Syst. Evol. Microbiol..

[9] Scholz M (2016). Strain-level microbial epidemiology and population genomics from shotgun metagenomics. Nat. Methods.

[10] Laver T (2015). Assessing the performance of the Oxford Nanopore Technologies MinION. Biomol. Detect. Quantif..

[11] Stern MJ, Ames GFL, Smith NH, Clare Robinson E, Higgins CF (1984). Repetitive extragenic palindromic sequences: a major component of the bacterial genome. Cell.

[12] Versalovic, J., Schneider, M. & Bruijn, F. De. Genomic fingerprinting of bacteria using repetitive sequence-based polymerase chain reaction. *Methods Mol. Cell. Biol*. **5**, 25–40 (1994).

[13] Versalovic J, Woods CR, Georghiou PR, Hamill RJ, Lupski JR (1993). DNA-based identification and epidemiologic typing of bacterial pathogens. Arch. Pathol. Lab. Med..

[14] Olive DM, Bean P (1999). Principles and applications of ligation mediated PCR methods for DNA-based typing of microbial organisms. J. Clin. Microbiol..

[15] De Vuyst L (2008). Validation of the (GTG)5-rep-PCR fingerprinting technique for rapid classification and identification of acetic acid bacteria, with a focus on isolates from Ghanaian fermented cocoa beans. Int. J. Food Microbiol..

[16] Gevers D, Huys G, Swings J (2001). Applicability of rep-PCR fingerprinting for identification of *Lactobacillus* species. FEMS Microbiol. Lett..

[17] Ishii S, Sadowsky MJ (2009). Applications of the rep-PCR DNA fingerprinting technique to study microbial diversity, ecology and evolution. Environ. Microbiol..

[18] Tafvizi F, Tajabadi Ebrahimi M (2015). Application of repetitive extragenic palindromic elements based on PCR in detection of genetic relationship of lactic acid bacteria species isolated from traditional fermented food products. J. Agric. Sci. Technol..

[19] Wise MG (2009). Predicting *Salmonella enterica* serotypes by repetitive sequence-based PCR. J. Microbiol. Methods.

[20] Nurhayati, Priyambada ID, Radjasa OK, Widada J (2017). Repetitive element palindromic PCR (Rep-PCR) as a genetic tool to study diversity in amylolytic bacteria. Adv. Sci. Lett..

[21] Healy M (2005). Microbial DNA typing by automated repetitive-sequence-based PCR. J. Clin. Microbiol..

[22] Koren S (2017). Canu: scalable and accurate long-read assembly via adaptive κ-mer weighting and repeat separation. Genome Res..

[23] Appuhamy S, Parton R, Coote JG, Gibbs HA (1997). Genomic fingerprinting of Haemophilus somnus by a combination of PCR methods. J. Clin. Microbiol..

[24] Woods CR, Versalovic J, Koeuth T, Lupski JR (1992). Analysis of relationships among isolates of Citrobacter diversus by using DNA fingerprints generated by repetitive sequence-based primers in the polymerase chain reaction. J. Clin. Microbiol..

[25] Harvey J, Norwood DE, Gilmour A (2004). Comparison of repetitive element sequence-based PCR with multilocus enzyme electrophoresis and pulsed field gel electrophoresis for typing Listeria monocytogenes food isolates. Food Microbiol..

[26] Clarridge JE (1995). Strategy to detect and identify Bartonella species in routine clinical laboratory yields *Bartonella henselae* from human immunodeficiency virus- positive patient and unique *Bartonella* strain from His cat. J. Clin. Microbiol..

[27] Gunawardana GA, Townsend KM, Frost AJ (2000). Molecular characterisation of avian *Pasteurella multocida* isolates from Australia and Vietnam by REP-PCR and PFGE. Vet. Microbiol..

[28] Northey G, Gal M, Rahmati A, Brazier JS (2005). Subtyping of *Clostridium difficile* PCR ribotype 001 by REP-PCR and PFGE. J. Med. Microbiol..

[29] Chou CH, Wang C (2006). Genetic relatedness between *Listeria monocytogenes* isolates from seafood and humans using PFGE and REP-PCR. Int. J. Food Microbiol..

[30] Mohapatra BR, Mazumder A (2008). Comparative efficacy of five different rep-PCR methods to discriminate *Escherichia coli* populations in aquatic environments. Water Sci. Technol..

[31] Hamon M, Bierne H, Cossart P (2006). Listeria monocytogenes: a multifaceted model. Nat. Rev. Microbiol..

[32] Leekitcharoenphon, P., Nielsen, E. M., Kaas, R. S., Lund, O. & Aarestrup, F. M. Evaluation of whole genome sequencing for outbreak detection of salmonella enterica. *PLoS ONE*10.1371/journal.pone (2014). 10.1371/journal.pone.0087991 PMC3913712 24505344

[33] Albufera U, Bhugaloo-Vial P, Issack MI, Jaufeerally-Fakim Y (2009). Molecular characterization of Salmonella isolates by REP-PCR and RAPD analysis. Infect. Genet. Evol..

[34] Konstantinidis KT, Tiedje JM (2005). Genomic insights that advance the species definition for prokaryotes. Proc. Natl Acad. Sci. USA.

[35] Konstantinidis KT, Ramette A, Tiedje JM (2006). Toward a more robust assessment of intraspecies diversity, using fewer genetic markers. Appl. Environ. Microbiol..

[36] Jacobsen A, Hendriksen RS, Aaresturp FM, Ussery DW, Friis C (2011). The *Salmonella enterica* Pan-genome. Microb. Ecol..

[37] Martin M (2011). Cutadapt removes adapter sequences from high-throughput sequencing reads. EMBnet J..

[38] Edgar RC (2010). Search and clustering orders of magnitude faster than BLAST. Bioinformatics.

[39] Kim D, Song L, Breitwieser FP, Salzberg SL (2016). Centrifuge: rapid and sensitive classification of metagenomic sequences. Genome Res..

[40] Wood Derrick E, Salzberg Steven L (2014). Kraken: ultrafast metagenomic sequence classification using exact alignments. Genome Biology.

[41] Mapleson, D., Accinelli, G. G., Kettleborough, G., Wright, J. & Clavijo, B. J. KAT: a K-mer analysis toolkit to quality control NGS datasets and genome assemblies. *Bioinformatics*10.1093/bioinformatics/btw663 (2017). 10.1093/bioinformatics/btw663 PMC5408915 27797770

[42] Bolger AM, Lohse M, Usadel B (2014). Trimmomatic: a flexible trimmer for Illumina sequence data. Bioinformatics.

[43] Rognes Torbjørn, Flouri Tomáš, Nichols Ben, Quince Christopher, Mahé Frédéric (2016). VSEARCH: a versatile open source tool for metagenomics. PeerJ.

[44] Bankevich A (2012). SPAdes: a new genome assembly algorithm and its applications to single-cell sequencing. J. Comput. Biol..

