# Supplementary material for: DNA enrichment and tagmentation method for species-level identification and strain-level differentiation using ON-rep-seq
Source: Commun Biol. 2019 Oct 10;2:369. doi: 10.1038/s42003-019-0617-x (PMC6787052; doi:10.1038/s42003-019-0617-x)
Supplement: Supplementary file 1 — Supplementary Information [file 42003_2019_617_MOESM1_ESM.pdf]

Supplementary Figures

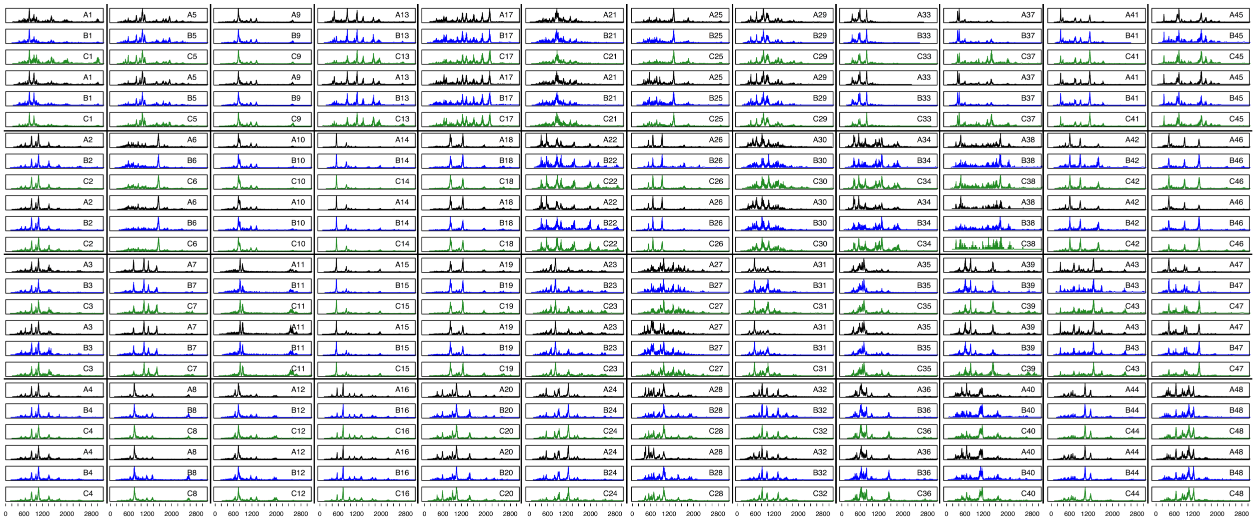

replicate	bacterium	strain	replicate	bacterium	strain
A1 B1 C1	*Streptoccocus thermophilus*	S0	A25 B25 C25	*Lactobacillus amylovorus*	DSM20531T
A2 B2 C2	*Salmonella enterica* subsp. enterica serovar Typhimurium	u292	A26 B26 C26	*Lactobacillus acidophilus*	LMG 9433T, T30
A3 B3 C3	*Salmonella enterica* subsp. enterica serovar Typhimurium	4//74	A27 B27 C27	*Escherichia coli*	DSM1058
A4 B4 C4	*Salmonella enterica* subsp. enterica serovar Typhimurium	C5	A28 B28 C28	*Bifidobacterium pseudocatenulatum*	LMG10505
A5 B5 C5	*Salmonella enterica* subsp. enterica serovar Oranienburg	0112F	A29 B29 C29	*Bifidobacterium longum subsp. infantis*	DSM20090
A6 B6 C6	*Pediococcus pentosaceus*	DSM20336T	A30 B30 C30	*Bifidobacterium longum*	LMG13196
A7 B7 C7	*Pediococcus claussenii*	DSM14800T	A31 B31 C31	*Bifidobacterium catenulatum*	LMG11043
A8 B8 C8	*Listeria monocytogenes*	EGDe	A32 B32 C32	*Bifidobacterium breve*	DSM20091
A9 B9 C9	*Listeria monocytogenes*	L028	A33 B33 C33	*Bifidobacterium bifidum*	LMG11041
A10 B10 C10	*Listeria monocytogenes*	N53-1	A34 B34 C34	*Bifidobacterium animalis*	DSM10140
A11 B11 C11	*Listeria monocytogenes*	12067	A35 B35 C35	*Bifidobacterium adolescentis*	DSM20083
A12 B12 C12	*Listeria monocytogenes*	42222/180	A36 B36 C36	*Bacteroides thetaiotaomicron*	DSM2079
A13 B13 C13	*Leuconostoc mesenteroides mesenteroides*	DSM20343T	A37 B37 C37	*Bacteroides fragilis*	DSM2151
A14 B14 C14	*Lactococcus lactis* subsp. *cremoris*	MG1363	A38 B38 C38	*Bacteriodes vulgatus*	LMG17263
A15 B15 C15	*Lactococcus lactis* subsp. *cremoris*	Wg2	A39 B39 C39	*Bacteriodes thethaiotaomicron*	DSM2079
A16 B16 C16	*Lactobacillus sakei* subsp. *sakei*	DSM20017T	A40 B40 C40	*Bacteriodes intestinalis*	DSM17393
A17 B17 C17	*Lactobacillus rhamnosus*	DSM20021T	A41 B41 C41	*Bacteriodes finegoldii*	DSM17565
A18 B18 C18	*Lactobacillus plantarum*	ATCC14917T, LLFH15	A42 B42 C42	*Bacteriodes eggerthii*	DSM20697
A19 B19 C19	*Lactobacillus plantarum*	DSM20174T	A43 B43 C43	*Bacteriodes cellulosilyticus*	DSM14838
A20 B20 C20	*Lactobacillus paracasei* subsp. *paracasei*	NCFB151T, LLFH13	A44 B44 C44	*Bacillus subtilis*	own
A21 B21 C21	*Lactobacillus fermentum*	DSM20052T	A45 B45 C45	*Bacillus lichniformis*	CMG19409
A22 B22 C22	*Lactobacillus paracasei*	own	A46 B46 C46	*Bacillus cereus*	15
A23 B23 C23	*Lactobacillus casei*	DSM20011T, LLFH4	A47 B47 C47	*Bacillus cereus*	38 (GR177)
A24 B24 C24	*Lactobacillus brevis*	GGUC30670T, LLFH24	A48 B48 C48	*Akkermansia muciniphila*	DSMZ 22959

Supplementary Figure 1. LCp generated for 48 bacterial isolates using Oxford Nanopore Technology based rep-PCR amplicon sequencing (ON-rep-seq). The black, blue and green profiles indicate data collected during run A, B and C respectively for which each technical replicate received different barcode. All isolates were analysed in duplicates within each run. The list of bacterial taxa matching given LCp is given in the table.

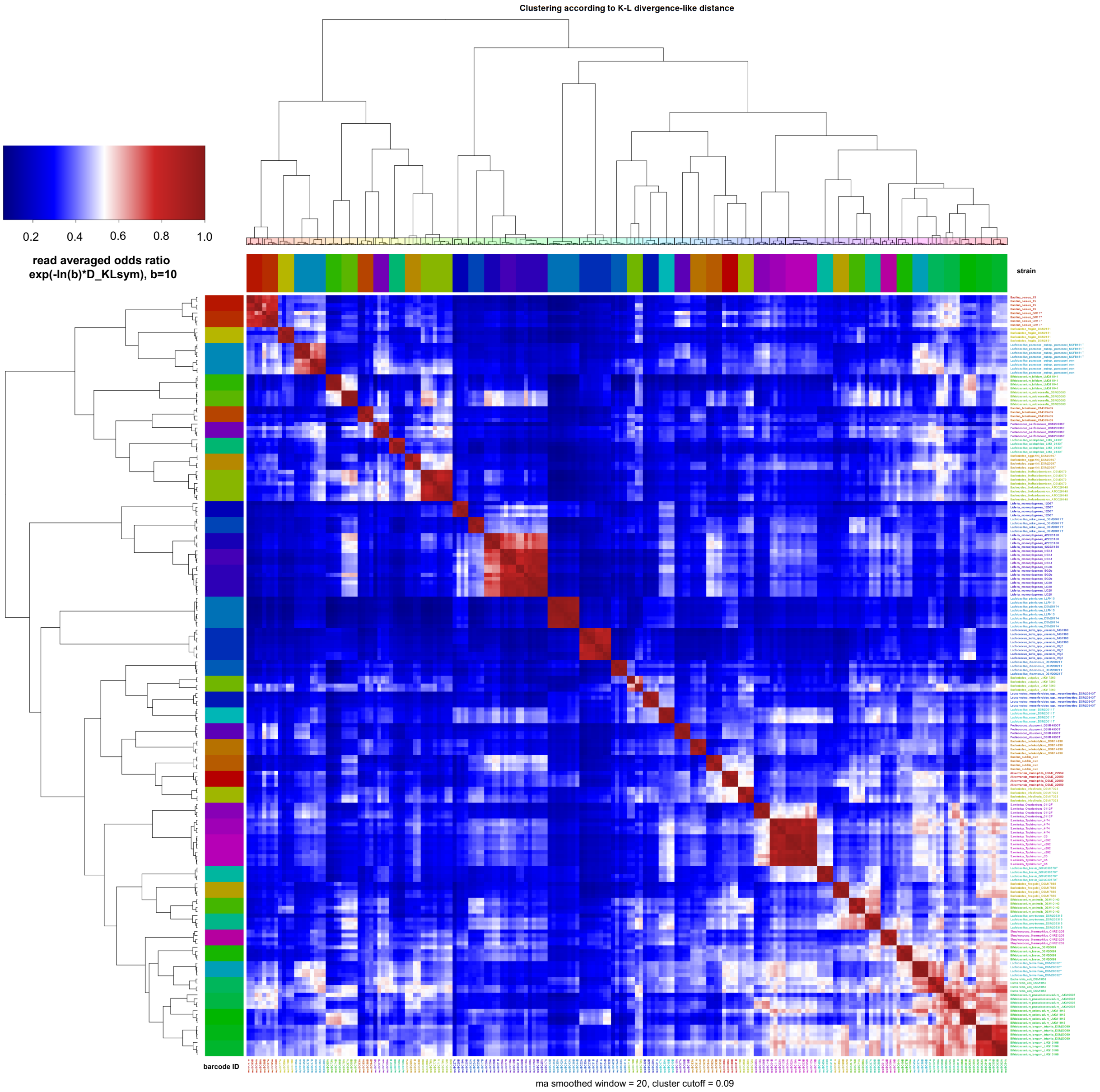

Supplementary Figure 2. Row/Column clustering according to "Ward.D2" hierarchical clustering on D_KLsym distance of all 48 isolates. Heatmap showing similarity (exp(-ln(b)*D_KLsym), b=10), and clustering according to cutoff=0.09. The detailed analysis using varying cutoff value (no single cutoff achieves exact separation between all and only different LCp, see Supplementary Figure 4 C, D ROC curves) and LCp visual inspection allowed for accurate differentiation between all except two pairs of bacterial strains described thoroughly in the results section (see Figure 3 and Supplementary figure 2 for details). Technical replicates from the third run “repC” were removed from the analysis due to higher short/long reads imbalance.

A

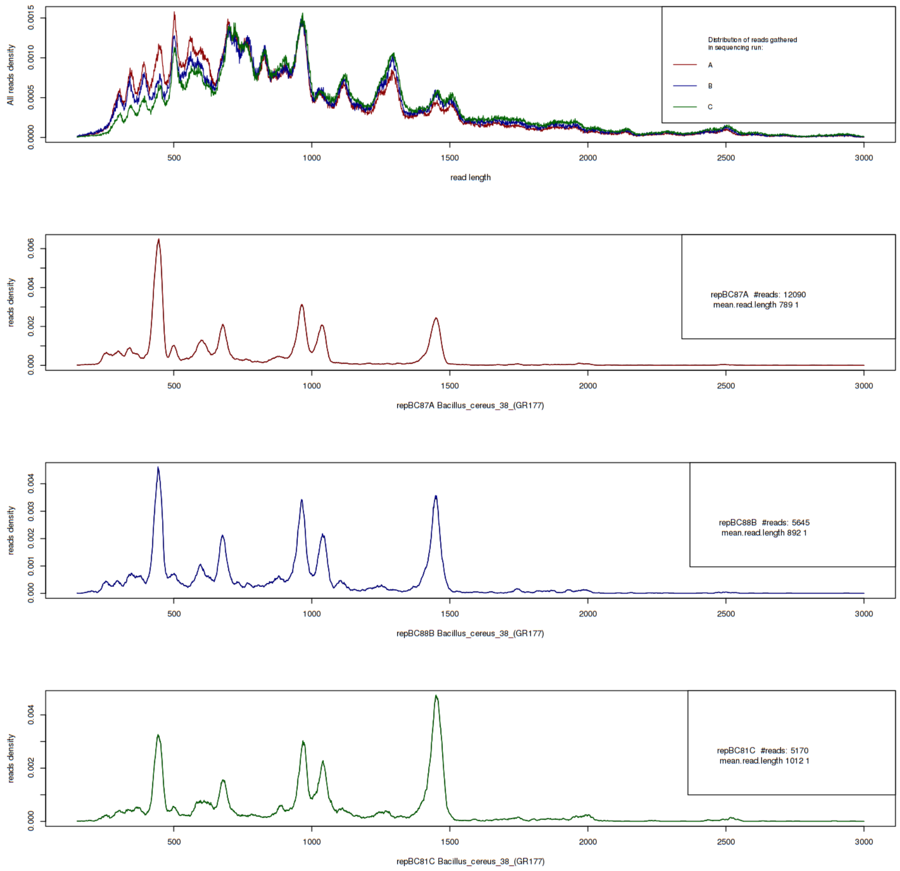

B

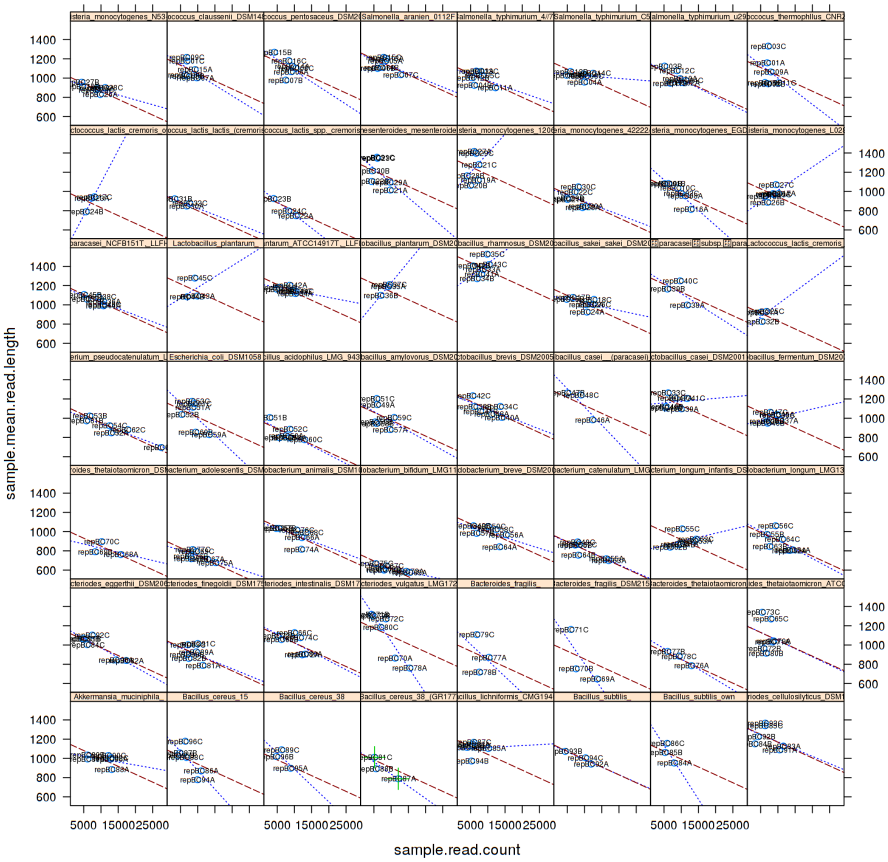

Supplementary Figure 3

A) Top panel presents distribution of lengths of reads obtained in 3 separate consecutive sequencing runs A, B, C on the same flow cell. Third run C obtained less short reads, some differences are also visible in second run B, compared to the first run A. Bottom 3 panels show LCps of Bacillus_cereus_38 (GR177) strain obtained from runs A,B,C.

B) Regression analysis of mean read length from LCp vs read count in LCp, data shown in separate panels for each strain replicates. Red dashed line is regression line obtained in all samples analysis, blue lines are regression lines for each strain only. Green markers mark runs A, C for Bacillus_cereus_38(GR177) (panels 2 and 4 in A).

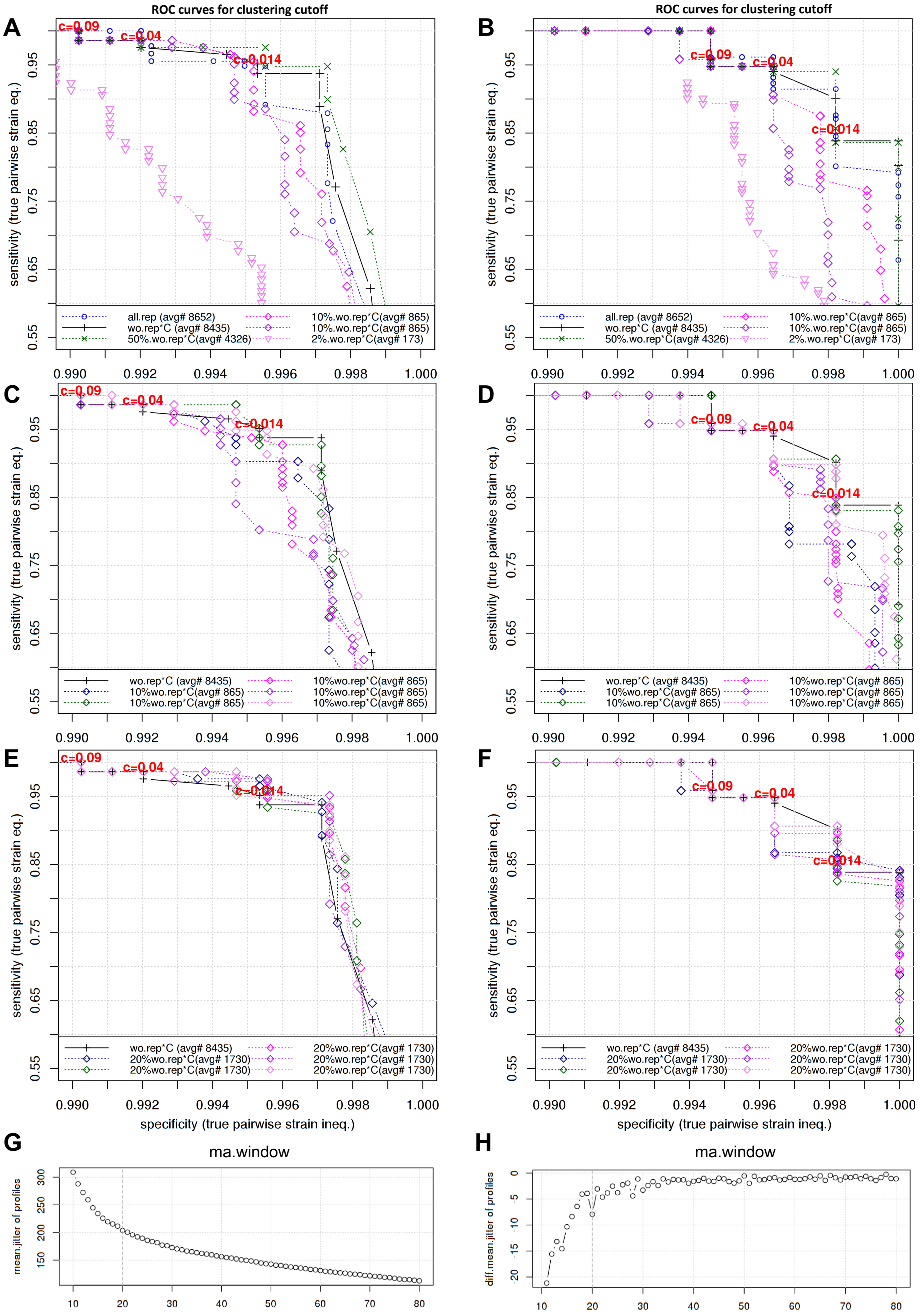

Supplementary Figure 4. Peaks profiles comparison

A-F) Receiver operating characteristic (ROC) curves of pairwise "same/not-the-same" strain discrimination in various cutoffs c (diff. step=0.005), for various subsets of data: "all", "wo.rep*C" dataset without the third sequencing run "C" on twice used flow cells, "50%.wo.rep*C" subsample half the size of original, "20%.wo.rep*C" five subsamples 1/20th of reads, "10%.wo.rep*C" seven subsamples 1/10th of reads and "2%.wo.rep*C" 1/50th of reads. On x-axis specificity, the percentage of correctly identified "not-the-same strain" pairs out of all such pairs (36096 for wo.rep*C), on y-axis sensitivity, the percentage of correctly identified "same strain" pairs, out of all such pairs (768 for wo.rep*C). A) Clustering according to sample strain label, viewed as a whole method performance, in contrast to B. B) Clustering according to sample strain similarity derived from visual inspection of profiles, thus these curves correspond more to D_KLsym-based profile comparison performance, than to the whole method. Values on the plot: c=0.09 (sp 0.9947, se 0.9583), c=0.014 (sp 0.9982, se 0.8490). All cutoffs "c" values marked for "wo.rep*C". C) Clustering according to sample strain label using 5 iterations of 10% subsets. D) Clustering according to sample strain similarity derived from visual inspection of profiles using 5 iterations of 20% subsets. The analysis shows that 20% subsets perform similarly to the whole dataset what indicates the theoretical throughput of ON-rep-seq to range from 960 (for ~1.5M reads) to 1440 (for ~2.5M reads) isolates per flow cell. The analysis on panels E), F) shows that 20% subsets perform similarly to the whole dataset, what indicates the theoretical throughput of ON-rep-seq to range from 960 (for ~1.5M reads) to 1440 (for ~2.5M reads) isolates per flow cell. G) mean jitter of all profiles dependence on smoothing moving average "ma" window size. Jitter was defined as an average number of times when profile's discrete derivative changes sign (change to 0 was counted as 0.5). H) discrete derivative (diff lag=1) of the (top) mean jitter. Sizes of ma.window > 20 change mean jitter slowly and steadily suggestive of stabilization (noise decoupling) of information content in higher smoothing window results.

Supplementary Tables

Supplementary Table 1. Identification of MLST genes alleles among selected strains of *Salmonella enterica* subsp. *enterica* serovar Typhimurium and *Listeria monocytogenes*

Strain	Gene	Allele type	Strain	Gene	Allele type
Salmonella enterica U292	*aroC*	10	*Listeria monocytogenes* EDGe	*abcZ*	*6*
	dnaN	7		*bglA*	*5*
	hemD	12		*cat*	*6*
	hisD	9		***dapE***	***20***
	purE	5		*dat*	*176*
	sucA	9		*ldh*	*4*
	thrA	2		*lhk*	*1*
Salmonella enterica C5	*aroC*	10	*Listeria monocytogenes* L028	*abcZ*	*6*
	dnaN	7		*bglA*	*5*
	hemD	12		*cat*	*6*
	hisD	9		***dapE***	***51***
	purE	5		*dat*	*176*
	sucA	9		*ldh*	*4*
	thrA	2		*lhk*	*1*
Salmonella enterica 4/74	*aroC*	10			
	dnaN	7			
	hemD	12			
	hisD	9			
	purE	5			
	sucA	9			
	thrA	2			

Supplementary Table 2. Details regarding benchmarking of two R9.4.1 flow cells.

	**Flow cell 1**				**Flow cell 2**		
**Run ID**	**A**	**B**	**C**	**D**	**A**	**B**	**C**
Run Time (h)	4	4	4	4	4	4	12
Break between the next run (day)	1	4	3	7	1	1	1
Active pores at start	1347	1324	1098	925	1034	779	615
Voltage at start (mV)	-180	-180	-190	-195	-180	-180	-190
Initial sequences in strand	~300	~200	~150	~50	~200	~120	~70
Total number of high quality reads collected	9.4×10^5	7.9×10^5	5.7×10^5	2.2×10^5	10.5×10^5	5.7×10^5	8.7×10^5
Library concentration loaded in 12 µl (ng/ µl)	2.5	1.8	3.0	1.6	3.2	2.1	2.4

Both flow cells generated in total similar amount of data, although flow cell 1 was in much better condition and had more active pores at start what allowed to perform four consecutive runs. Flow cell 2 had lower number of active pores at arrival and seemed to deteriorate faster therefore only three runs were conducted. Last run was elongated to 12 h in order to collect maximum amount of data from declining flow cell. The data from the first benchmarked flow cell were used solely to test the optimal concentration of DNA needed and viability of the flow cell while data from the second flow cell are presented herein
