# Supplementary material for: DNA enrichment and tagmentation method for species-level identification and strain-level differentiation using ON-rep-seq
Source: Commun Biol. 2019 Oct 10;2:369. doi: 10.1038/s42003-019-0617-x (PMC6787052; doi:10.1038/s42003-019-0617-x)
Supplement: Supplementary file 2 — Description of additional supplementary files [file 42003_2019_617_MOESM2_ESM.docx]

**Supplementary Data 1: List of 96 barcodes for bacterial isolate amplicon tagmentation with ON-rep-seq**

The list of 96 Oxford Nanopore Technologies (ONT) compatible adapters given in 5’-3’ orientation. The 15bp spacer separating ONT motor protein adapter from the barcode sequence and (GTG)5 pairing region. The spacer was added to ensure higher tolerance for the low-quality at the beginning of the sequence entering the pore and thus higher recovery of barcode sequence. At the same time the spacer sequence was designed to prevent creations of stem-loops in relatively long primers during low temperature annealing step.
