# Supplementary material for: DNA enrichment and tagmentation method for species-level identification and strain-level differentiation using ON-rep-seq
Source: Commun Biol. 2019 Oct 10;2:369. doi: 10.1038/s42003-019-0617-x (PMC6787052; doi:10.1038/s42003-019-0617-x)
Supplement: Supplementary file 3 — Supplementary Data 1 [file 42003_2019_617_MOESM3_ESM.docx]

**Supplementary Table 3. List of 96 barcodes for bacterial isolate Rep-PCR amplicon tagmentation.**

| name | spacer ONbarcode 5x(GTG) |
| --- | --- |
| Rep-BC01 | **GTCTCGTCCGCTCGG** CACAAAGACACCGACAACTTTCTT **GTGGTGGTGGTGGTG** |
| Rep-BC02 | **GTTAGTTGATGTAGT** ACAGACGACTACAAACGGAATCGA **GTGGTGGTGGTGGTG** |
| Rep-BC03 | **GTCTCGTCCGCTCGG** CCTGGTAACTGGGACACAAGACTC **GTGGTGGTGGTGGTG** |
| Rep-BC04 | **GTTAGTTGATGTAGT** TAGGGAAACACGATAGAATCCGAA **GTGGTGGTGGTGGTG** |
| Rep-BC05 | **GTCTCGTCCGCTCGG** AAGGTTACACAAACCCTGGACAAG **GTGGTGGTGGTGGTG** |
| Rep-BC06 | **GTTAGTTGATGTAGT** GACTACTTTCTGCCTTTGCGAGAA **GTGGTGGTGGTGGTG** |
| Rep-BC07 | **GATATGATATAGATA** AAGGATTCATTCCCACGGTAACAC **GTGGTGGTGGTGGTG** |
| Rep-BC08 | **GTTAGTTGATGTAGT** ACGTAACTTGGTTTGTTCCCTGAA **GTGGTGGTGGTGGTG** |
| Rep-BC09 | **GTCTCGTCCGCTCGG** AACCAAGACTCGCTGTGCCTAGTT **GTGGTGGTGGTGGTG** |
| Rep-BC10 | **GTTAGTTGATGTAGT** GAGAGGACAAAGGTTTCAACGCTT **GTGGTGGTGGTGGTG** |
| Rep-BC11 | **GTTAGTTGATGTAGT** TCCATTCCCTCCGATAGATGAAAC **GTGGTGGTGGTGGTG** |
| Rep-BC12 | **GTCTCGTCCGCTCGG** TCCGATTCTGCTTCTTTCTACCTG **GTGGTGGTGGTGGTG** |
| Rep-BC13 | **GTCTCGTCCGCTCGG** TCACACGAGTATGGAAGTCGTTCT **GTGGTGGTGGTGGTG** |
| Rep-BC14 | **GTTAGTTGATGTAGT** TCTATGGGTCCCAAGAGACTCGTT **GTGGTGGTGGTGGTG** |
| Rep-BC15 | **GTTAGTTGATGTAGT** CAGTGGTGTTAGCGAGGTAGACCT **GTGGTGGTGGTGGTG** |
| Rep-BC16 | **TACATTGATGCATGG** AGTACGAACCACTGTCAGTTGACG **GTGGTGGTGGTGGTG** |
| Rep-BC17 | **GTCTCGTCCGCTCGG** ATCAGAGGTACTTTCCTGGAGGGT **GTGGTGGTGGTGGTG** |
| Rep-BC18 | **GTTAGTTGATGTAGT** GCCTATCTAGGTTGTTGGGTTTGG **GTGGTGGTGGTGGTG** |
| Rep-BC19 | **GTTAGTTGATGTAGT** ATCTCTTGACACTGCACGAGGAAC **GTGGTGGTGGTGGTG** |
| Rep-BC20 | **GTTAGTTGATGTAGT** ATGAGTTCTCGTAACAGGACGCAA **GTGGTGGTGGTGGTG** |
| Rep-BC21 | **GTTAGTTGATGTAGT** TAGAGAACGGACAATGAGAGGCTC **GTGGTGGTGGTGGTG** |
| Rep-BC22 | **GTTAGTTGATGTAGT** CGTACTTTGATACATGGCAGTGGT **GTGGTGGTGGTGGTG** |
| Rep-BC23 | **GTCTCGTCCGCTCGG** CGAGGAGGTTCACTGGGTAGTAAG **GTGGTGGTGGTGGTG** |
| Rep-BC24 | **GTTAGTTGATGTAGT** CTAACCCATCATGCAGAACTATGC **GTGGTGGTGGTGGTG** |
| Rep-BC25 | **GTCTCGTCCGCTCGG** CATTGCGTTGCATACCCAACTTAC **GTGGTGGTGGTGGTG** |
| Rep-BC26 | **TACATTGATGCATGG** ATGAGAATGCGTAGTCGCTGTATG **GTGGTGGTGGTGGTG** |
| Rep-BC27 | **GTCTCGTCCGCTCGG** TGTAAGAGGTGAATCTAACCGTCG **GTGGTGGTGGTGGTG** |
| Rep-BC28 | **GTTAGTTGATGTAGT** GATACGGTGCCTTCTTAGGTTTCA **GTGGTGGTGGTGGTG** |
| Rep-BC29 | **GTTAGTTGATGTAGT** GGTCTGTCAACCCAAGGTGTCTAG **GTGGTGGTGGTGGTG** |
| Rep-BC30 | **GTTAGTTGATGTAGT** TGGGTCGAAGTAGATCCTCACTGA **GTGGTGGTGGTGGTG** |
| Rep-BC31 | **GTCTCGTCCGCTCGG** CAATGTAACTGATTGCTGTACGCA **GTGGTGGTGGTGGTG** |
| Rep-BC32 | **GTTAGTTGATGTAGT** ATGACGTTGTCGGACTTCTACTGG **GTGGTGGTGGTGGTG** |
| Rep-BC33 | **GTCTCGTCCGCTCGG** AGTTACCCAACCGTACCAAGTCTG **GTGGTGGTGGTGGTG** |
| Rep-BC34 | **GTTAGTTGATGTAGT** GCCTTTGACTTGAGTTCTTCGTCC **GTGGTGGTGGTGGTG** |
| Rep-BC35 | **GTCTCGTCCGCTCGG** GCAGTCCCTCAGCTTCGTAAGTAG **GTGGTGGTGGTGGTG** |
| Rep-BC36 | **GTTAGTTGATGTAGT** TGTTTCCTCCTCTAACTGGGACAT **GTGGTGGTGGTGGTG** |
| Rep-BC37 | **GTCTCGTCCGCTCGG** TGATACTAAGCATCAATCGCAAGC **GTGGTGGTGGTGGTG** |
| Rep-BC38 | **GTTAGTTGATGTAGT** TTCTCTGTATCGTCCTCCTGTGGT **GTGGTGGTGGTGGTG** |
| Rep-BC39 | **GTTAGTTGATGTAGT** GAGAGGCTCTAGTTGACACTGTGG **GTGGTGGTGGTGGTG** |
| Rep-BC40 | **GTTAGTTGATGTAGT** GGCTATCCTTGGTCATCCAAACTA **GTGGTGGTGGTGGTG** |
| Rep-BC41 | **GTTAGTTGATGTAGT** CGTGTACTTCTCTGGACGAACTCC **GTGGTGGTGGTGGTG** |
| Rep-BC42 | **GTCTCGTCCGCTCGG** CTGGCAGGTATGCCTTACACGTAG **GTGGTGGTGGTGGTG** |
| Rep-BC43 | **GTTAGTTGATGTAGT** CTACCGTCGAGTCAACAACGAAAG **GTGGTGGTGGTGGTG** |
| Rep-BC44 | **GTTAGTTGATGTAGT** GAGTGGGAAGGAACCCTTTCTACT **GTGGTGGTGGTGGTG** |
| Rep-BC45 | **GTCTCGTCCGCTCGG** CACTGAAGGCATCTCTGTTGGATC **GTGGTGGTGGTGGTG** |
| Rep-BC46 | **GTTAGTTGATGTAGT** CAGGAGAATGAAGTGGAACACAGC **GTGGTGGTGGTGGTG** |
| Rep-BC47 | **GTCTCGTCCGCTCGG** GAACTACCTGTGGGAAAGTTGCAC **GTGGTGGTGGTGGTG** |
| Rep-BC48 | **GTTAGTTGATGTAGT** TACAGGTGTACCACGTTCCAGATG **GTGGTGGTGGTGGTG** |
| Rep-BC49 | **GTCTCGTCCGCTCGG** CTAGATGTTCAAAGCTGCACCAGT **GTGGTGGTGGTGGTG** |
| Rep-BC50 | **GTTAGTTGATGTAGT** ACGCAGGAAGTTACCAAAGTCCAT **GTGGTGGTGGTGGTG** |
| Rep-BC51 | **GTCTCGTCCGCTCGG** GAGGACCCAGTAGGCTCATTCAAC **GTGGTGGTGGTGGTG** |
| Rep-BC52 | **GTTAGTTGATGTAGT** GTCCACGAACAATCTTGTCTCTCA **GTGGTGGTGGTGGTG** |
| Rep-BC53 | **GTCTCGTCCGCTCGG** CTTTGCATGAGACGGTCTGAATCT **GTGGTGGTGGTGGTG** |
| Rep-BC54 | **GTTAGTTGATGTAGT** CATGCTCCTTAGTCAAAGCTCTTG **GTGGTGGTGGTGGTG** |
| Rep-BC55 | **GTCTCGTCCGCTCGG** CGTAGATCAGGGTCTCATCTTCCA **GTGGTGGTGGTGGTG** |
| Rep-BC56 | **GTCTCGTCCGCTCGG** TTCATGCCACCTGTTGAGTAGTGA **GTGGTGGTGGTGGTG** |
| Rep-BC57 | **TACATTGATGCATGG** ACTTCCGAAGGAGATTGACCTAGC **GTGGTGGTGGTGGTG** |
| Rep-BC58 | **GTTAGTTGATGTAGT** TCAGACTCACGGAGGAGTAACCTG **GTGGTGGTGGTGGTG** |
| Rep-BC59 | **GTTAGTTGATGTAGT** ACCTTGCTTTCCCTTCTTGATTGA **GTGGTGGTGGTGGTG** |
| Rep-BC60 | **GTTAGTTGATGTAGT** CCATAGAAGCCTTGGTTGAACATG **GTGGTGGTGGTGGTG** |
| Rep-BC61 | **TACATTGATGCATGG** GTGCTGAGGCACATAGTACCCTCT **GTGGTGGTGGTGGTG** |
| Rep-BC62 | **GTTAGTTGATGTAGT** TACGTCCTGAAGTAAGTGTGGGTG **GTGGTGGTGGTGGTG** |
| Rep-BC63 | **GTTAGTTGATGTAGT** GTTCAAGACCCAGGAACTTCAGAA **GTGGTGGTGGTGGTG** |
| Rep-BC64 | **GTTAGTTGATGTAGT** GAAAGTCGATGAACGGTGTCTGTC **GTGGTGGTGGTGGTG** |
| Rep-BC65 | **GTCTCGTCCGCTCGG** CCTTGTCTGGAGGAAGACTGAGAA **GTGGTGGTGGTGGTG** |
| Rep-BC66 | **GTCTCGTCCGCTCGG** GAAGTTAGAAGCCACAAGGATCGG **GTGGTGGTGGTGGTG** |
| Rep-BC67 | **TACATTGATGCATGG** GGTGAGCACACGAGTATGACAAAC **GTGGTGGTGGTGGTG** |
| Rep-BC68 | **GTCTCGTCCGCTCGG** CCACCTTCGTGTTTGCTTAGATTC **GTGGTGGTGGTGGTG** |
| Rep-BC69 | **GTTAGTTGATGTAGT** AGATCACATGAGGCTCGGACTGTA **GTGGTGGTGGTGGTG** |
| Rep-BC70 | **GTTAGTTGATGTAGT** ACACTCCATTCGTAGGATCTCGGT **GTGGTGGTGGTGGTG** |
| Rep-BC71 | **GTCTCGTCCGCTCGG** CTGTTACTACCTGATGCTCCCAGG **GTGGTGGTGGTGGTG** |
| Rep-BC72 | **GTTAGTTGATGTAGT** GTCGGTATGGAAGACAGTCAGCTA **GTGGTGGTGGTGGTG** |
| Rep-BC73 | **GTCTCGTCCGCTCGG** GAGGGTTCTGTCATCCTGTTTCTT **GTGGTGGTGGTGGTG** |
| Rep-BC74 | **GTTAGTTGATGTAGT** AGTGGAAGTGTTGGGATGCTTGTA **GTGGTGGTGGTGGTG** |
| Rep-BC75 | **GTCTCGTCCGCTCGG** ACAACAGGGTTCATCACAATGGTC **GTGGTGGTGGTGGTG** |
| Rep-BC76 | **GTTAGTTGATGTAGT** GTCCAGGGTTGATGTAACAAGCAT **GTGGTGGTGGTGGTG** |
| Rep-BC77 | **GTCTCGTCCGCTCGG** GTTGTATCCCTGAGAAACAGGTCG **GTGGTGGTGGTGGTG** |
| Rep-BC78 | **GTTAGTTGATGTAGT** TTCTGATTCAAAGGTTCGGTTGTT **GTGGTGGTGGTGGTG** |
| Rep-BC79 | **GTCTCGTCCGCTCGG** CAGCAGTGAGAACTATCTCCGAGA **GTGGTGGTGGTGGTG** |
| Rep-BC80 | **GTTAGTTGATGTAGT** GAATCGCTATCCTATGTTCATCCG **GTGGTGGTGGTGGTG** |
| Rep-BC81 | **GTCTCGTCCGCTCGG** CCGAAACAACTTCACAAGATGAGG **GTGGTGGTGGTGGTG** |
| Rep-BC82 | **GTTAGTTGATGTAGT** TAGTCCTGGAACTCGACATACCGT **GTGGTGGTGGTGGTG** |
| Rep-BC83 | **GTCTCGTCCGCTCGG** TTCGACCTTACCTAGATCAAGCCA **GTGGTGGTGGTGGTG** |
| Rep-BC84 | **GTTAGTTGATGTAGT** TGGCACAGGTTCTAGGTCCACTAC **GTGGTGGTGGTGGTG** |
| Rep-BC85 | **GTCTCGTCCGCTCGG** GATCATCCAACTAACTCCTCCGTT **GTGGTGGTGGTGGTG** |
| Rep-BC86 | **GTCTCGTCCGCTCGG** TACTTACGCTTGTTGGGATCACCT **GTGGTGGTGGTGGTG** |
| Rep-BC87 | **GTCTCGTCCGCTCGG** CCTCCCTAACAACAGGAGCATGTA **GTGGTGGTGGTGGTG** |
| Rep-BC88 | **GTTAGTTGATGTAGT** CTGCTTCGGATCGGTAGTAGAAGA **GTGGTGGTGGTGGTG** |
| Rep-BC89 | **GTCTCGTCCGCTCGG** CAACTAGCCAAACATTGATGCTGT **GTGGTGGTGGTGGTG** |
| Rep-BC90 | **GTTAGTTGATGTAGT** GCCTCAAACCGTACCCTCTACATC **GTGGTGGTGGTGGTG** |
| Rep-BC91 | **GTCTCGTCCGCTCGG** AGTAGCGTGAGTTCCTATGGAGCC **GTGGTGGTGGTGGTG** |
| Rep-BC92 | **GTCTCGTCCGCTCGG** GGTCCTGTATCTTTCCACTCACAA **GTGGTGGTGGTGGTG** |
| Rep-BC93 | **GTCTCGTCCGCTCGG** CCCAAGTCTGAAGTGATGGAAACT **GTGGTGGTGGTGGTG** |
| Rep-BC94 | **GTTAGTTGATGTAGT** GTAGGTGGCAGTTTGAGGACAATC **GTGGTGGTGGTGGTG** |
| Rep-BC95 | **GTCTCGTCCGCTCGG** AAGTCCATTCTTCTTCCAGACAGG **GTGGTGGTGGTGGTG** |
| Rep-BC96 | **GTCTCGTCCGCTCGG** ATGGTGGACTCTATGACCGTTCAG **GTGGTGGTGGTGGTG** |
