# Supplementary material for: DNA enrichment and tagmentation method for species-level identification and strain-level differentiation using ON-rep-seq
Source: Commun Biol. 2019 Oct 10;2:369. doi: 10.1038/s42003-019-0617-x (PMC6787052; doi:10.1038/s42003-019-0617-x)
Supplement: Supplementary file 4 — Reporting Summary [file 42003_2019_617_MOESM4_ESM.pdf]

nature research

Corresponding author(s): Lukasz Krych

Last updated by author(s): 06-09-2019

Reporting Summary

Nature Research wishes to improve the reproducibility of the work that we publish. This form provides structure for consistency and transparency in reporting. For further information on Nature Research policies, see Authors & Referees and the Editorial Policy Checklist.

Statistics

For all statistical analyses, confirm that the following items are present in the figure legend, table legend, main text, or Methods section.

n/a	Confirmed	
☐	☒	The exact sample size (n) for each experimental group/condition, given as a discrete number and unit of measurement
☐	☒	A statement on whether measurements were taken from distinct samples or whether the same sample was measured repeatedly
☒	☐	The statistical test(s) used AND whether they are one- or two-sided<br>*Only common tests should be described solely by name; describe more complex techniques in the Methods section.*
☒	☐	A description of all covariates tested
☒	☐	A description of any assumptions or corrections, such as tests of normality and adjustment for multiple comparisons
☐	☒	A full description of the statistical parameters including central tendency (e.g. means) or other basic estimates (e.g. regression coefficient) AND variation (e.g. standard deviation) or associated estimates of uncertainty (e.g. confidence intervals)
☒	☐	For null hypothesis testing, the test statistic (e.g. F, t, r) with confidence intervals, effect sizes, degrees of freedom and P value noted<br>*Give P values as exact values whenever suitable.*
☒	☐	For Bayesian analysis, information on the choice of priors and Markov chain Monte Carlo settings
☐	☒	For hierarchical and complex designs, identification of the appropriate level for tests and full reporting of outcomes
☒	☐	Estimates of effect sizes (e.g. Cohen's d, Pearson's r), indicating how they were calculated

Our web collection on statistics for biologists contains articles on many of the points above.

Software and code

Policy information about availability of computer code

Data collection	MinKNOW 1.10.23
Data analysis	Guppy 2.1.3; Porechop 0.2.2; Canu 1.6; VSEARCH 2.13.6; kraken2 2.0.7-beta; see for details: Zenodo repository (DOI 10.5281/zenodo.3384842)

For manuscripts utilizing custom algorithms or software that are central to the research but not yet described in published literature, software must be made available to editors/reviewers. We strongly encourage code deposition in a community repository (e.g. GitHub). See the Nature Research guidelines for submitting code & software for further information.

Data

Policy information about availability of data

All manuscripts must include a data availability statement. This statement should provide the following information, where applicable:

- Accession codes, unique identifiers, or web links for publicly available datasets
- A list of figures that have associated raw data
- A description of any restrictions on data availability

ON-rep-seq pipeline is available at: bitbucket.org/modelscat/on-rep-seq
Fastq files can be downloaded from SRA NCBI repository (#SUB4333515)

Field-specific reporting

Please select the one below that is the best fit for your research. If you are not sure, read the appropriate sections before making your selection.

☒ Life sciences ☐ Behavioural & social sciences ☐ Ecological, evolutionary & environmental sciences

For a reference copy of the document with all sections, see nature.com/documents/nr-reporting-summary-flat.pdf

Life sciences study design

All studies must disclose on these points even when the disclosure is negative.

Sample size	We have developed a method for bacterial DNA enrichment that includes barcoding (tagmentation with DNA ologonucleotides).<br>In theory it would be enough to demonstrate the performance of the method using a single model bacterium (e.g. E.coli) and about 12-24 barcodes (standard in majority of available barcoding kits). In our study we have used 48 bacterial isolates to demonstrate applicability of the method to type more organisms. We have designed 96 barcodes to ensure bulk analysis using standard laboratory plate format.
Data exclusions	In order to provide the details regarding flow cell benchmark 2 independent flow cells were used. However, only samples on one flow cell were designed to carry necessary amount of technical replicates and repetitions therefore solely data from this flow cell were used in the manuscript whereas data generated with the first flow cell were used only to demonstrate technical parameters of the run. The exclusion of the dataset to demonstrate the performance of the entire method was pre-established.
Replication	The whole analysis was performed in six technical replicates and 3 independent runs which extend the standard use of 2 technical replicates in most studies. We have performed the whole analysis three times to demonstrate the reproducibility of the entire procedure.
Randomization	Randomization was not required in this study as method did not required comparison to a control group nor composed of separated batches. However, we did perform rotation of the DNA barcodes tested, meaning that each bacterial isolate out of 6 technical replicates had a different barcode.
Blinding	Blinding was not required in this study as no bias could be expected from testers' performance during the analysis.

Reporting for specific materials, systems and methods

We require information from authors about some types of materials, experimental systems and methods used in many studies. Here, indicate whether each material, system or method listed is relevant to your study. If you are not sure if a list item applies to your research, read the appropriate section before selecting a response.

Materials & experimental systems

n/a	Involved in the study	
☒	☐	Antibodies
☒	☐	Eukaryotic cell lines
☒	☐	Palaeontology
☒	☐	Animals and other organisms
☒	☐	Human research participants
☒	☐	Clinical data

Methods

n/a	Involved in the study	
☒	☐	ChIP-seq
☒	☐	Flow cytometry
☒	☐	MRI-based neuroimaging
